# Effectiveness of blending E-learning with field trip on Chinese herbal medicine education: quasi-experimental study

**DOI:** 10.1186/s12906-020-03034-y

**Published:** 2020-08-10

**Authors:** Lei Li, Chi Wing Tam, Ning Wang, Fan Cheung, Qing Zhou, Cheng Zhang, Chien-shan Cheng, Lei Xiong, Yibin Feng

**Affiliations:** 1 School of Chinese Medicine, Li Ka Shing Faculty of Medicine, The University of Hong Kong, 10 Sassoon Road, Pokfulam, Hong Kong SAR, People’s Republic of China; 2 Yunnan University of Chinese Medicine, 1076 Yuhua Road, Chenggong District, Kunming, 650500 Yunnan Province People’s Republic of China

**Keywords:** Chinese herbal, Medical education, Blended learning, E-learning, Materia Medica, Medicinal plants, Teaching, Moodle, Field trip

## Abstract

**Background:**

Chinese Medicine education is part of professional medical training in Hong Kong. An important element of this is herbal medicine, which requires both theoretical and practical knowledge. A field trip programme was adopted to provide students with direct experience of medicinal plants studied in lectures. However, problems with the current programme were identified in learning outcome assessment and long-term knowledge management. To improve the teaching quality, a Moodle e-learning module was designed for augmentation. This study aimed to quantitatively evaluate the effectiveness of the Moodle module in supplementing the current field trip programme.

**Methods:**

Prospective quasi-experiment. Participants were 49 year-2 students in the Bachelor of Chinese Medicine programme. A Moodle module including five online activities regarding two groups of herbal plants was integrated before and after the field trip. Fill-in-the-blank questions were used to assess the learning outcome. Also, a questionnaire was developed to collect student feedback as the secondary outcome.

**Results:**

For herbal plants in Group A, the assessment score was higher in Moodle group (29.65 ± 5.0) than for the control group (21.65 ± 6.5) (*P* <  0.01). For herbal plants in Group B, the assessment score was higher for the Moodle group (28.68 ± 4.7) than for the control group (24.26 ± 7.7) (*P* <  0.01). The questionnaire results showed that students were satisfied with the Moodle platform.

**Conclusions:**

A specially designed Moodle module may be effective in augmenting the field trip for Chinese herbal medicine education.

## Background

### Chinese herbal medicine education

Professional health care training in Hong Kong, as in China generally, requires university students in medical discipline to be equipped with some basic knowledge and skills in conventional and integrative medicine, particularly Chinese medicine (CM) [1]. This training in CM focuses not only on the basic theory and clinical practice of CM, but also on recognizing Chinese medicinal herbs by their appearance in the wild and in CM dispensary, because herbal medicine is the major treatment method in CM. A fundamental course called “Chinese Materia Medica” is currently offered to higher education students in medicine-related disciplines to provide a solid foundation of knowledge on herbal medicine. In our school, “Chinese Materia Medica” is held in Semester 1 (September to December) of the second academic year. It is conducted mainly in a lecture-based form as in traditional instructional design of most universities. While factual knowledge of origins and parts of medicinal plants used are covered, the emphasis of teaching and assessment remains the properties, indications, route of administration and dosage, adverse effects as well as the identification of prepared herbs. Lecturing and demonstration of specimens have limits in cultivating essential skills in macroscopic identification, as well as thorough understanding of properties of herbs.

### Prior work

Experiential learning, defined as “learning from life experience” [2], is one potential strategy to address limitations in didactic learning. Experiential learning has been reported to be widely used in professional programmes [3–6], but its effectiveness has not been proven rigorously in the literature [4], especially in the context of CM education. In our school, field trips are employed as a mean of experiential learning. A field trip is a journey that allows students and teachers to leave the classroom to experience settings away from their normal environment [7]. Field trips are sometimes conducted in medical programmes as a direct approach to teaching and learning essential knowledge and activities in medical practice [8–11]. The studied field trip programme is held in Summer Semester to allow students to recognize and directly in contact with the raw Chinese herbs in the wild that they have previously studied in the “Chinese Materia Medica” course. In addition to this direct experience, the field trip offers students the opportunity to learn about the social and cultural background of CM [7]. Both the direct experience and the deepening of background knowledge achieved through experiential learning can help students to deepen the understanding of Chinese herbs obtained in lectures, facilitating a reinforcement loop between experimental learning and lecture-based learning. Despite the appealing benefits inherent to experiential learning, several problems in the current field trip were identified based on feedback on the course design of “Chinese Materia Medica” and its field trip component. Students requested improvements in the learning facilities and assessment approaches of the course. On the other hand, teachers found it difficult to evaluate the quality of students’ participation in the field trip. This was mainly due to the relatively low teacher-to-student ratio, and students tend to scatter around in the field. Teachers were also concerned about whether the expected outcomes (experience and knowledge) were effectively delivered to each student during the field trip. Additionally, students had difficulty reviewing what they have learned during the field trip because the trip required experiencing indigenous Chinese medical herbs in the wild under natural conditions in Mainland China, meaning that the related perceptions and knowledge could be only provided on-site during the field trip. Finally, it was not feasible to evaluate students’ learning outcomes after the field trip. As a result, the knowledge management of this field trip programme as a resource for the school and university was poor, and the teachers, tutors, and students had difficulty providing feedback regarding their respective positions in this system, which limits the establishment of standard assessments of the output of the field trip study programme. There is an urgent need for an improved design of the CM field trip course.

### Goal of this study

As the use of information technology has advanced over the past few decades, e-learning, also known as web-based learning, has gained increasing popularity. Many curricula designed for people of different ages have adopted e-learning as a teaching method [12–14]. E-learning has also been integrated into the field of medical education, from pre-licensure to continuing [15, 16]. Evidence suggests that e-learning is efficient in terms of influencing students’ knowledge, skills, and attitudes when used appropriately [16, 17]. Moodle is a popular learning management system that is promoted in our university as an online e-learning platform. Educators can use Moodle without charge to create online learning sites with Internet access. Moodle brings a flexible learning environment to education, and it can adapt to our needs. Individual course sites can be designed using different Moodle modules, including assignments, forums, quizzes, surveys, chat rooms, and workshops [18–20]. Many educators and researchers have explored creative ways with Moodle to build powerful and effective online courses [18, 19, 21–23]. Importantly, Moodle is compatible with mobile devices, so students can access the material anytime and anywhere.

It was hypothesised that blending e-learning with field trip can achieve better learning outcomes. This study aimed to evaluate the effectiveness of e-learning in augmenting teaching and learning activities of the field trip programme in CM professional training. Undergraduate students in the Bachelor of Chinese Medicine (BChinMed) programme were able to review and being evaluated on what they learned during the field trip online, together with records of their activities on Moodle, teachers and tutors involved could better assess students’ learning outcome. A quasi-experimental design [24] was used to study the impact of Moodle on the learning outcomes of CM students in terms of recognizing medicinal plants after the field trip. This study was conducted in the School of Chinese Medicine at the University of Hong Kong.

## Material and methods

### Participants and setting

In the School of Chinese Medicine at the University of Hong Kong, field trip study programmes have been used for more than 10 years. The field trip is a compulsory summer course for second-year students in the BChinMed programme. The field trip is usually conducted in June, when students have finished the “Chinese Materia Medica” course. The location of the field trip is Kunming in Yunnan Province, which is well known as the “Kingdom of Plants”. A briefing session is held 1 week before the field trip to introduce the activities and requirements of the course. During the field trip, students study herbal plants in the wild under the guidance of teachers who are specialists in herbal plant identification. To complete the requirements of the field trip, students must participate in all the activities arranged by the School and complete the assignments requested by the teachers.

A total of 49 s-year BChinMed students, including 26 students in a 5-year programme and 23 in a 6-year programme, participated in a 6-day field trip programme in Yunnan in June 2014. Because the students had completed the “Chinese Materia Medica” course in the previous semester, they possessed some prior knowledge about recognizing wild herbal plants. Convenience sampling was used, students were divided into two groups according to their study programme, namely a 5-year cohort (*n* = 26) and a 6-year cohort (*n* = 23).

### Study design

In this study, a prospective quasi-experimental design was adopted. To identify any influences of differences between the students, students’ gender and prior knowledge of the course content were collected as baseline characteristics (Table 1). Two measures were used to assess prior knowledge of related content: 1) scores obtained from the students’ recent “China Materia Medica” examination (out of 100 marks); and 2) scores on a 15-min baseline test completed 1 week before the field trip, in the form of fill-in-the-blank questions including 15 randomly selected images of herbal plants (out of 15 marks). A brief introduction to using Moodle was given to students 1 week before the field trip to ensure that technical capacity in working with Moodle would not impact the test scores.

**Table 1:** Pre-teaching data

	5-year cohort	6-year cohort	*P* value
Number of students	26	23	
Gender			
Men	10	12	0.396
Women	16	11	
Recent “Chinese Materia Medica” examination score (out of 100) (mean ± SD)	75.18 ± 8.19	70.69 ± 7.89	0.869
Baseline test score (out of 15) (mean ± SD)	0.17 ± 0.4	0.10 ± 0.3	0.204

Sixty-four herbal plants that students encountered during the field trip were selected for fill-in-the-blank questions in the assessment. The 64 herbal plants were randomly divided into two groups: Group A and Group B, with 32 plants in each group.

The students in the 5-year cohort studied the herbal plants in Group A during the field trip and used the Moodle platform to review after the field trip (experiment intervention). Herbal plants in Group B were delivered only during the field trip (active control). On the other hand, students in the 6-year cohort studied the herbal plants in Group B during the field trip and used the Moodle platform to review after the field trip (experiment intervention). Herbal plants in Group A were delivered only during the field trip (active control). In the introduction session before the field trip, students had been informed that 100 herbs would be tested after the field trip such that students should not only study herbs available on Moodle review.

**Fig. 1:**
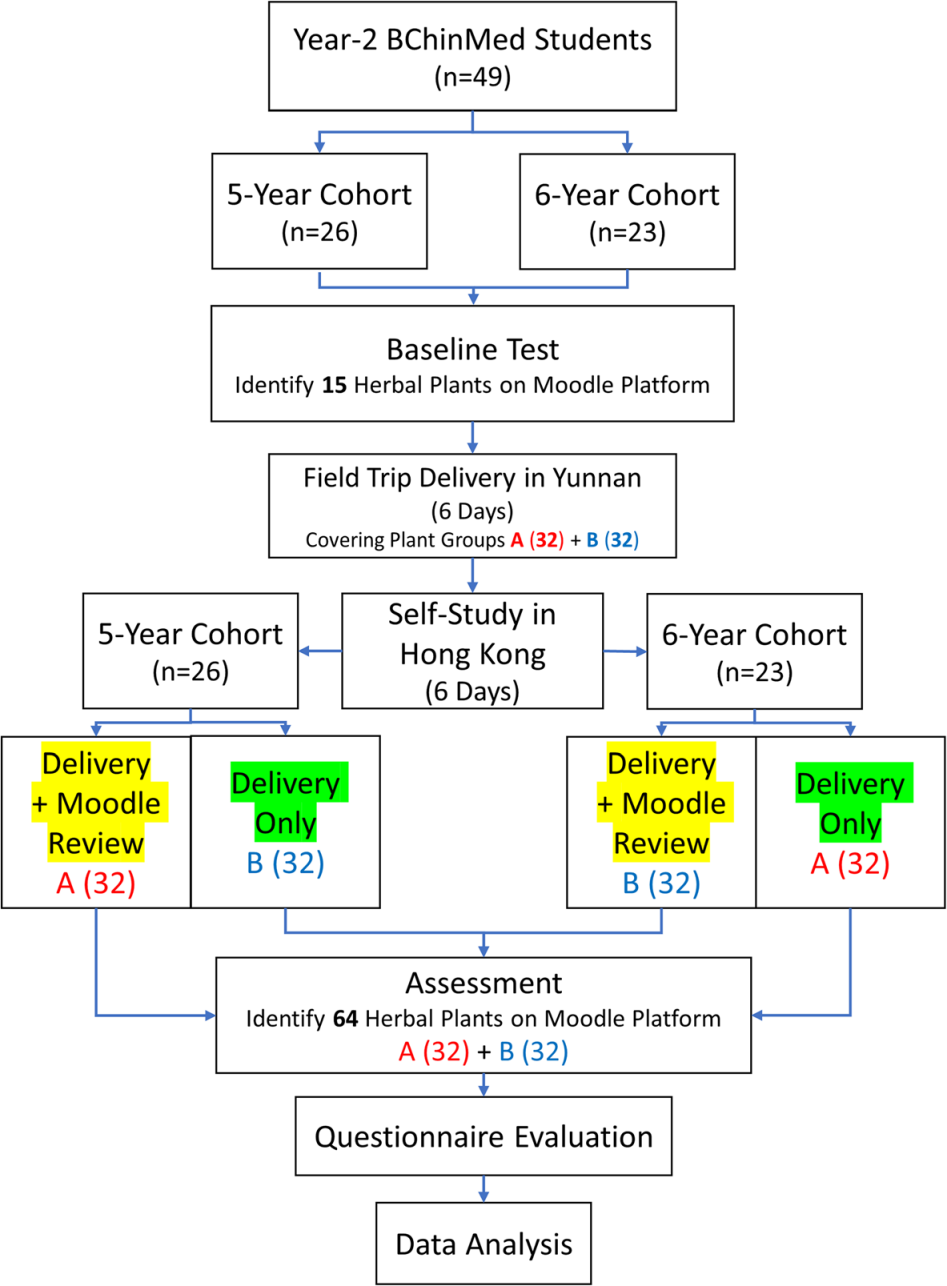
Study design. Flowchart of the research design

After the field trip and 6 days of Moodle-enhanced self-study, students completed a fill-in-the-blank assessment via Moodle to identify the 64 images of herbal plants. The primary outcome for data analysis is the score of the assessment (the correct number of herbal plants identified). Figure 1 shows the flowchart of the research design.

### Moodle E-learning platform design

The Moodle course site was established and evaluated by teaching staff members at the School of Chinese Medicine. Technicians in the e-Learning Pedagogical Support Unit revised the course site to improve the feasibility and accessibility of the teaching material according to feedback from staff members. The online Moodle course site was divided into five parts:

#### Section 1: course review

This section provided course information and preparation material for the field trip. The names of 100 herbal plants that students might encounter during the Yunnan field trip were listed in the handbook with sketched pictures and written descriptions. The handbook could be downloaded and used during the on-site study, for example to identify herbal plants based on the provided pictures and taking notes during the field trip. Students were able to access this section 1 week before the briefing session.

#### Section 2: warm-up test (baseline test)

Fill-in-the-blank questions on the identification of 15 herbal plants were provided in this section. Documented photos from previous field trips were used here. All of the students completed the warm-up test during the briefing session. Scores on the warm-up test were considered the baseline test result of the study. The warm-up test was used only to evaluate students’ knowledge of herbal plant identification before the field trip—not for course assessment.

#### Section 3: atlas of herbal plants

The atlas included a list of 100 commonly used herbal plants with high-quality photos of fresh plants and corresponding photos of dried herbs, as well as a description of each herb. Documented photos from previous field trips were used in this section.

#### Section 4: field trip herbal photo sharing

A herbal photo sharing database was set up and improved based on feedback after testing. In this section, students could upload their herbal photos collected during the field trip and add descriptions. Teachers and other students were able to post comments on each photo.

#### Section 5: herbal plant identification exercise

In this section, three sets of herbal plant identification exercises were provided. These “drag-and-drop” exercises were designed and improved by teachers at the School of Chinese Medicine and technicians in the eLearning Pedagogical Support Unit. Documented photos from previous field trips were used in this section.

All 49 students were given access to the Moodle e-learning platform after they returned from the field trip. The students could log in to Moodle to review herbal photos and perform self-test exercises at any time during the 6-day self-study period. Moodle could be accessed via computer or any mobile device. Teachers, tutors and technical staff can view students’ activity logs in the control panel.

### Post-teaching assessment

The Moodle-assisted learning outcome was assessed using a quiz administered right after the last day of self-study, in the form of a recognition test. The two-hour quiz consisted of 64 fill-in-the-blank questions (each carrying two marks) on tutor-collected photos of herbs. These questions assessed three cognitive levels of learning, including the ability to recall the particular appearance of medicinal plants, the ability to comprehend the basic characters of different plants, and the ability to apply the skill of plant recognition on the test. Students in the 5-year cohort had access to a Moodle database containing information on 32 herbs that were not included in the Moodle database of the 6-year cohort students, and vice versa. This allowed us to conduct an analysis of the learning outcome by statistically comparing the test scores of students who had access to information on particular herbs through Moodle with the scores of those did not have access to the Moodle information on these herbs.

### Questionnaire

A questionnaire was developed with face validity to evaluate the Moodle course site as a secondary outcome. This 12-item questionnaire evaluated five aspects: overall evaluation, Moodle user experience, activities in Moodle, learning efficacy, and future development. A five-point Likert scale evaluation system was used (5 = strongly agree, 4 = agree, 3 = neutral, 2 = disagree, 1 = strongly disagree). Questions are presented in Table 3 with the results. At the end of questionnaire, there was one box for collecting qualitative feedback. These self-administered questionnaires were distributed to all participants after the assessment.

### Data analysis

All data were entered into the Statistical Package for Social Sciences (SPSS version 22.0, IBM, Armonk, NY, USA) statistical analysis software. For the pre-teaching data, the chi-square test was used to analyse gender differences between the two cohorts. Students’ recent examination scores for the “Chinese Materia Medica” course, baseline test scores, and assessment scores were analysed using independent *t*-tests. The significance threshold was set at 0.05. The effect size was also calculated using Cohen’s *d* statistics.

## Results

All 49 students (27 women and 22 men) participated in the baseline test and briefing session, as well as the subsequent 6-day field trip. These students were then asked to complete a 6-day period of self-study on Moodle after returning to Hong Kong. Forty-nine questionnaires were distributed to the students along with the administration of post-teaching assessment after that. All students completed the post-teaching assessment. While two students finished the post-test without submitting the questionnaires, 47 questionnaires were returned, and 42 of these were complete and valid for analysis. The response rate was 85.7%.

### Pre-teaching data

All the students enrolled in the field trip had completed the “Chinese Materia Medica” course, where they received lectures about the features and recognition of medicinal plants. This background was assumed to provide the necessary knowledge base for the field trip programme. To eliminate confounding factors, we analysed demographic data on students in each group (Table 1). The gender ratio (male/female) was 10/16 in the 5-year cohort and 12/11 in the 6-year cohort. The “Chinese Materia Medica” course examination scores of the 5-year cohort students did not differ substantially from those of the 6-year cohort students. Because the examination was conducted in the previous semester and the scores might not reflect the current knowledge base of the students, a quiz on the content in the form of a recognition test was also completed by the students prior to the field trip. Fill-in-the-blank questions regarding 15 images of medicinal plants randomly selected from the pool of 64 images for the post-field trip assessment were used in this quiz. There was no significant difference in the baseline test scores of the two groups of students, indicating that students in both groups had minimal knowledge on recognizing medicinal plants in the wild. These data indicate that the students’ characteristics were not confounding variables in this study.

### Post-teaching assessment

The students in the 5-year cohort used Moodle to review the 32 herbal plants in Group A (intervention group), whereas the students in the 6-year cohort were not provided access to these review materials on Moodle (control group). For this group of plants, we found that the average score was significantly higher in the 5-year cohort (29.65 ± 5.0) than in the 6-year cohort (21.65 ± 6.5) (*P* <  0.01) (Table 2). As for the 32 herbal plants in Group B, the students in the 6-year cohort used Moodle to review these plants (intervention group), whereas the students in the 5-year cohort were not provided access to these Moodle review materials (control group). We found that the average score was significantly higher in the 6-year cohort (28.68 ± 4.7) than in the 5-year cohort (24.26 ± 7.7) (*P* <  0.01) (Table 2). In addition, we calculated the Cohen’s *d* for the effect size, which is 1.38.

**Table 2:** Post-teaching assessment results for herbal plant identification

	N	Score (out of 64) (mean ± SD)	*P* value
**Herbal plants in Group A**			
5-year cohort (intervention group)	26	29.65 ± 5.0	< 0.01
6-year cohort (control group)	23	21.65 ± 6.5	
**Herbal plants in Group B**			
5-year cohort (control group)	26	24.26 ± 7.7	< 0.01
6-year cohort (intervention group)	23	28.68 ± 4.7	

### Moodle access

**Fig. 2:**
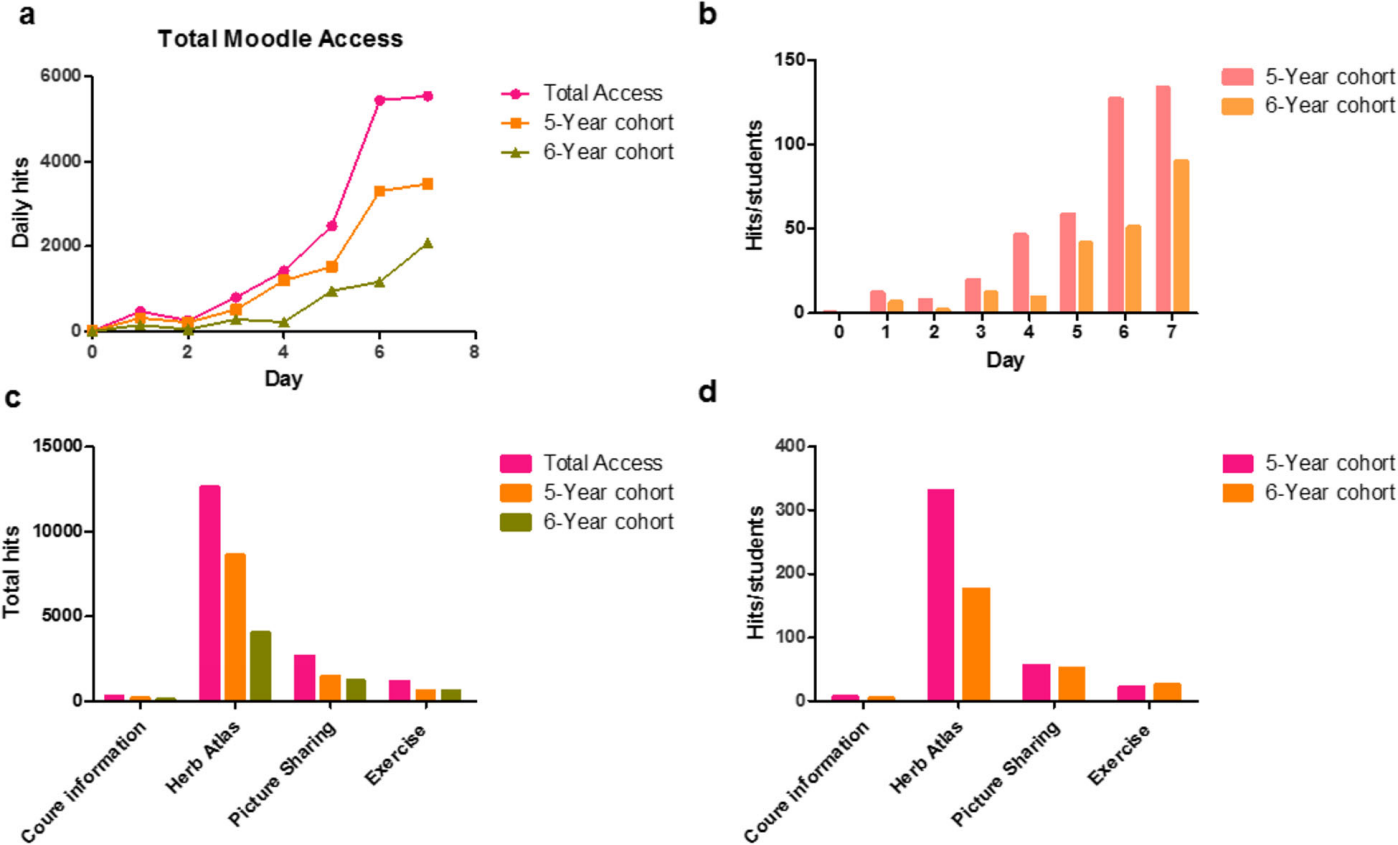
Statistics on student access to the Moodle system. **a** Total daily hits of the students’ Moodle activities; **b** Comparison of total daily hits per person of Moodle activities between students in the 5-year cohort and students in the 6-year cohort; **c** Moodle activity hits of all students; **d** Comparison of Moodle activity hits per person between students in the 5-year cohort and students in the 6-year cohort

Moodle recorded a total of 16,447 hits during the 6 days of review before the assessment. The daily Moodle hits for all 49 students are shown in Fig. 2a. A peak access to Moodle can be observed in the last 2 days before the assessment, when 5450 and 5551 hits were recorded, respectively. A comparison of daily Moodle hits between the 5-year cohort and the 6-year cohort is shown in Fig. 2b. More hits in terms of total and per-person access were recorded for the 5-year cohort’s Moodle access. Hits for the four activities in the Moodle e-learning platform were also recorded and compared between the two cohorts (Fig. 2c and d). The most frequently used content area was the herbal plant atlas (12,623 hits). The herbal photo sharing (2667 hits) and self-test exercises (1169 hits) were also frequently visited by the students. The 5-year cohort had about twice as many total hits for the herbal plant atlas, compared with the 6-year cohort. The total hits for herbal photo sharing and self-test exercises were nearly the same in the two cohorts.

### Questionnaire

The questionnaire statements and responses are shown in Table 3. The questionnaire results showed that about two-thirds of the students (66.6%) responded positively (strongly agree/agree) that the Moodle course site was effective in helping them to achieve the desired learning outcome of the field trip. Regarding the Moodle user experience, over half of the students commented that the Moodle e-learning platform was easy to navigate and use (61.9%), that the uploaded pictures and materials were useful (69%), and that they could cope with the Moodle course workload (66.6%). For the designed activities incorporated in Moodle, students appreciated the self-test exercises most; 71.4% of the students commented that this helped them to prepare for the test. Additionally, 66.7% of the students believed that the atlas of herbal plants helped them to systematically review what they had learned during the field trip, and 52.4% of the students responded that they liked to share their work on Moodle. The students also made positive comments about Moodle as useful for reviewing herbal plants studied during the field trip (71.4%), and they thought that it complemented the field trip learning well (69.1%). In terms of future development, students responded that they would review the herbal plant pictures after the course (57.2%), that they would recommend this Moodle course module to students participating in the following year’s field trip (57.1%), and that they would make use of this platform for further learning on herbal plant identification (59.5%).

**Table 3:** Summary of student responses to 12 statements in the questionnaire using a five-point Likert scale

Statement	Strongly agree		Agree		Neutral		Disagree		Strongly disagree		Mean ± SD
	N	%	N	%	N	%	N	%	N	%	
**Overall evaluation**											
1. The Moodle course was effective in helping me achieve the learning outcomes of BCHM2109/2606.	9	21.4	19	45.2	13	31	0	0	1	2.4	3.83 ± 0.9
**Moodle user experience**											
2. The uploaded pictures and materials were useful.	5	11.9	24	57.1	12	28.6	1	2.4	0	0	3.79 ± 0.7
3. It was easy to navigate and use.	8	19	18	42.9	15	35.7	1	2.4	0	0	3.79 ± 0.8
4. I was able to cope with the Moodle course site workload.	8	19	20	47.6	14	33.3	0	0	0	0	3.86 ± 0.7
**Activities in Moodle**											
5. The atlas of herbal plants helped me to systematically review what I had learned during the field trip.	7	16.7	21	50	10	23.8	4	9.5	0	0	3.74 ± 0.9
6. I like to share my work on Moodle.	6	14.3	16	38.1	19	45.2	1	2.4	0	0	3.64 ± 0.8
7. The self-test exercises helped me to prepare for the test.	9	21.4	21	50	11	26.2	1	2.4	0	0	3.90 ± 0.8
**Learning efficacy**											
8. The Moodle course site was useful for reviewing herbal plants studied during the field trip.	9	21.4	21	50	12	28.6	0	0	0	0	3.93 ± 0.7
9. The Moodle course site well complemented the field trip learning.	11	26.2	18	42.9	11	26.2	2	4.8	0	0	3.90 ± 0.8
**Future development**											
10. I will review the herbal plant photos even after the course.	7	16.7	17	40.5	15	35.7	3	7.1	0	0	3.67 ± 0.8
11. I will recommend this Moodle course site to students who will participate next year’s field trip.	10	23.8	14	33.3	13	31	4	9.5	1	2.4	3.67 ± 1.0
12. I will make use of this Moodle course site for further learning on herbal plant identification.	8	19	17	40.5	14	33.3	3	7.1	0	0	3.71 ± 0.9

Open-ended questions about students’ opinions on the Moodle e-learning platform were also recorded. Students commented that the best things about the Moodle e-learning platform were the “herbal plant atlas” and the “drag-and-drop exercises”, noting that it was “very useful and great for study”; “I can study the herbs at home and it’s quite useful”. Regarding the improvement of Moodle, students hoped that Moodle could provide more self-test exercises.

## Discussion

### Highlight

Many countries have introduced CM education programmes into their publicly funded higher education systems [25, 26]. Field trips provide experiential learning for students to learn outside the classroom, which is of great educational value in CM education. Particularly, by comparing the scores in the baseline test with the recent “Chinese Materia Medica” exam, the essentialness of field trip in herbal medicine education becomes obvious.

Feedback from teachers and students drives improvements in the learning and assessment approaches of the course. The incorporation of some e-learning elements into modern education on the “traditional” Chinese medicine was a timely pursuit. This study evaluated the effectiveness of a specially designed Moodle module blended with a field trip programme in CM education. To our knowledge, this was the first study to use a quantitative quasi-experimental design in an attempt to show the influence of e-learning in enhancing experiential learning by medical training on integrative medicine. Many studies have indicated that online courses have positive impacts on learning outcomes in medical education in particular settings, especially when blended [15, 16, 27]. Likewise, our results show that combining Moodle e-learning with the field trip enhanced students’ learning outcomes on herbal plant identification. The mixed effect of repeated interactive exercise with multimedia elements may play a role in refreshing students’ memory. Therefore, e-learning should be considered in similar field trip programmes, especially for post-trip review.

Another benefit of the Moodle module examined in this study is that students could access the e-learning platform using any mobile device at any time. Most students have access to a personal computer and feel comfortable using computer-based information resources in their learning. This is advantageous, because this means of learning is not limited by location or time, which was reflected in the students’ comments.

### Limitations

There are a few limitations that must be addressed in this quasi-experimental research. Firstly, convenience sampling may introduce selection bias, the study population may not represent all undergraduate students studying CM, due to factors such as subculture. Despite its lack of randomness, it allowed the study to be more feasible since contamination in the field trip and Moodle self-study would be remarkable if classmates in the same cohort were assigned to different interventions. Moreover, because of educational purposes, neither students nor investigators were blinded, observer effect might exist.

Other factors such as temporal effect and maturation effect were controlled by baseline test and timely assessment. Testing effect was minimized by using only 15 stock photos in the baseline test.

One may argue that the Moodle course containing Group A herbal plants assigned to students may act merely as reinforcement of short-term memory of selected herbs. In this study, all the 100 herbal plants were expected to be tested after the field trip by students. However, a third test in their commencement of next academic year may be considered to observe long term knowledge retention, with a month’s time free of any interventions.

Besides, scores in the post-teaching assessment were not high as expected. It could be due to the holiday mood in summer that hinders students’ motivation to revise. Another reason is that only “novice” but not “competence” stage [28] could be achieved through a single field trip, indicating the need for more opportunities. Apparently, higher number of hit counts were recorded in the 5-year cohort, suggesting more engagement that conflicts the former explanation. However, outliers may account for such hit counts. Meanwhile, scores of intervention group in both cohorts were close. It is likely reflecting the diminishing marginal returns of engagement and/or issues in learning efficiency. Also, lower scores of control group in 6-year cohort implicate the possibility of difference in difficulty of questions. Further investigations are required for supporting these explanations, with more advanced learner analytics [29].

Furthermore, the mean recent “Chinese Materia Medica” exam score in 5-year cohort were 5 points higher, despite statistically insignificance. Although unlikely, there might still be potential confounding between two groups of students.

In the 12-item questionnaire, “neutral” constituted 23.8–45.2% of responses. Under such sample size constraint, distortion effect to avoid extreme responses becomes considerable [30]. According to literature, participants choosing “neutral” are “non-attitude” and “undecided” due to different reasons [30]. Seven-point Likert scales may be more favourable in such setting for its potential in higher sensitivity [31].

Finally, in terms of the identification of herbal plants, virtual e-learning cannot replace the on-site field trip. During these field trips, students learn to identify herbal plants by sight as well as by touch, smell, and even taste. However, providing pictures in the Moodle e-learning platform allows visual recognition only. Therefore, the Moodle e-learning platform should be considered complementary to the learning achieved during the field trip.

## Conclusion

To conclude, blending a specially designed e-learning module with field trip can effectively enhance students’ achievements in recognising herbal plants as the learning outcome. Students’ frequent access to the module and results of questionnaire suggested that most of them were satisfied with the new module combining the on-site field trip with online Moodle learning. However, causation cannot be fully established due to potential threats of validity. Hence, such blending is a potentially beneficial approach in teaching Chinese Herbal Medicine.

## Data Availability

The datasets generated and/or analysed during the current study are not publicly available due to individual privacy but are available from the corresponding author on reasonable request.

## Abbreviations

CM: Chinese Medicine
BChinMed: Bachelor of Chinese Medicine

## References

[1] Chen HY, Feng Y, Lao L (2014). Chinese integrative medicine: inclusion of a Chinese medicine programme in a conventional medical institute. J Integr Med.

[2] Kolb DA (2015). Experiential learning: experience as the source of learning and development.

[3] Hill B (2017). Research into experiential learning in nurse education. Br J Nurs.

[4] Morris TH. Experiential learning – a systematic review and revision of Kolb’s model. Interact Learn Environ. 2019:1–14. 10.1080/10494820.2019.1570279.

[5] Yang YM, Kim CH, Briones MA, Hilinski JA, Greenwald M (2014). Instinctive clinical teaching: erasing the mental boundary between clinical education and patient care to promote natural learning. J Grad Med Educ.

[6] Tanaka K, Son D (2019). Experiential learning for junior residents as a part of community-based medical education in Japan. Educ Prim Care.

[7] Behrendt M, Franklin T (2014). A review of research on school field trips and their value in education. Int J Environ Sci Educ.

[8] Friedland AR, Rintel-Queller HC, Unnikrishnan D, Paul DA (2012). Field trips as a novel means of experiential learning in ambulatory pediatrics. J Grad Med Educ.

[9] Chang AY, Bass TL, Duwell M, Berger JS, Bangalore R, Lee NS (2017). The impact of "see the City you serve" field trip: an educational tool for teaching social determinants of health. J Grad Med Educ.

[10] Hartman M, Thomas S, Ayoob A (2018). Radiology field trips-a list of "must sees" in the radiology Department for Medical Students: how we do it. Acad Radiol.

[11] Claramita M, Setiawati EP, Kristina TN, Emilia O, van der Vleuten C (2019). Community-based educational design for undergraduate medical education: a grounded theory study. BMC Med Educ.

[12] Garrison DR. E-learning in the 21st century: a framework for research and practice. 2nd ed: GB. New York: Taylor & Francis Ltd - M.U.A; 2011.

[13] Clark RC (2016). E-learning and the science of instruction: proven guidelines for consumers and designers of multimedia learning. 4th ed. Mayer RE, editor.

[14] Rodrigues H, Almeida F, Figueiredo V, Lopes SL (2019). Tracking e-learning through published papers: a systematic review. Comput Educ.

[15] Walsh K (2013). Oxford textbook of medical education.

[16] Vaona A, Banzi R, Kwag KH, Rigon G, Cereda D, Pecoraro V (2018). E-learning for health professionals. Cochrane Database Syst Rev.

[17] Cook DA, Garside S, Levinson AJ, Dupras DM, Montori VM (2010). What do we mean by web-based learning? A systematic review of the variability of interventions. Med Educ.

[18] Rice WH, Moore M, Bailye M (2008). Moodle 1.9 e-learning course development [electronic resource] : a complete guide to successful learning using Moodle 1.9.

[19] Stanford J, Baker A, Wright C (2009). Moodle 1.9 for second language teaching [electronic resource] : engaging online language-learning activities using the Moodle platform.

[20] Cole JR (2008). Using Moodle.

[21] Kats Y (2013). Learning management systems and instructional design : best practices in online education.

[22] Purnama F, Usagawa T. Using real-time online preprocessed mouse tracking for lower storage and transmission costs. J Big Data. 2020;7(1):1–22. 10.1186/s40537-020-00304-x.

[23] Lakhal S, Mukamurera J, Bédard M-E, Heilporn G, Chauret M. Features fostering academic and social integration in blended synchronous courses in graduate programs. Int J Educ Technol High Educ. 2020;17(1):1–22. 10.1186/s41239-020-0180-z.

[24] Mills GE (2015). Educational research: competencies for analysis and applications, global edition.

[25] Karpa K (2012). Development and implementation of an herbal and natural product elective in undergraduate medical education. BMC Complement Altern Med.

[26] Quartey NK, Ma PH, Chung VC, Griffiths SM (2012). Complementary and alternative medicine education for medical profession: systematic review. Evid Based Complement Alternat Med.

[27] Liu Q, Peng W, Zhang F, Hu R, Li Y, Yan W (2016). The effectiveness of blended learning in health professions: systematic review and meta-analysis. J Med Internet Res.

[28] Mukhtar M, Gunderman RB (2017). The sixth stage: mastery. Acad Radiol.

[29] Maren S, Hendrik D, Slavi S, Marcus S (2014). Quality indicators for learning analytics. J Educ Technol Soc.

[30] Nadler JT, Weston R, Voyles EC (2015). Stuck in the middle: the use and interpretation of mid-points in items on questionnaires. J Gen Psychol.

[31] Finstad K (2010). Response interpolation and scale sensitivity: evidence against 5-point scales. J Usability Stud.

